# Book Reviews

**Published:** 1821-09

**Authors:** 


### CRITICAL ANALYSES

OF

### RECENT PUBLICATIONS, IN THE DIFFERENT BRANCHES OF MEDICINE AND SURGERY.

"I would have men know, that, though I reprehend the easie passing over of the causes of things "by ascribing them to secret and hidden vertues and properties; (for this hath arrested and laid "asleepe all true enquiry and indications;) yet I doe not understand but that, in the practical "part of knowledge, much will be left to experience and probation, whereunto indication cannot "so fully reach: and this not only in *specie*, but in *individuo*. Yet it was well said, *Vere scire* "*esse per causas scire*."—BACON.

*Essays on the Female Economy.* 1. *On the Periodical Discharge of the Human Female; with new Views of its Nature, Causes, and Influence on Disease: to which are added, Directions for its Management in the different Stages of Life.* 2. *On a Species of Abortion, not heretofore described, to which delicate Females in high Life are peculiarly liable; with a Mode of Treatment which has secured a happy Termination of the Pregnancy, where previously repeated Disappointment had been experienced.* By JOHN POWER, M.D. Physician-Accoucheur to the Westminster Lying-in Institution; Member of the Royal Medical Society of Edinburgh; Lecturer on Midwifery; and Author of "a Treatise on Midwifery, developing new Principles which tend materially to lessen the Sufferings of the Patient, and shorten the Duration of Labour, &c. &c." 8vo. pp. 101. Burgess and Hill, London.

THIS book will, we believe, give ample occasion to the author for lamenting that he has not, in this instance at least, been somewhat more mindful of the precepts of Horace respecting works which are the creations of the human imagination. Had he suffered the delight which we suppose he experienced on his fancy giving birth to what he may regard as brilliant thoughts, to have subsided but a little, he would, perhaps, have perceived what strange, indeed absurd, hypotheses he has here exposed to the scrutiny of public opinion: he would have seen from what erroneous data he has proceeded, and how much his arguments are wanting in sound logic; and he would have feared the influence such productions must have on his reputation as a practitioner of that branch of the medical art which requires so especially the qualities of correct observance and cool judgment in those who pretend to fulfil its very important duties.

With regard to the first of the subjects of which Dr. Power here treats,—that of menstruation in woman,—he does not, it is true, "presume to flatter himself that the view which he has taken, notwithstanding it appears [to himself] to be well supported by many well-established facts, will be deemed to afford a perfect and unobjectionable explication;" and we readily allow that hypotheses may be of eminent utility in physiology, and that, where we cannot attain to theories, plausible conjectures should be received with complacency : but there is a wide diversity between such considerations and the strange and visionary notions which constitute Dr. Power's hypothesis of menstruation; and when he remarks that, on the subject just mentioned, "an infinity of conjectures, the offsprings of hypothesis, have hitherto supplied the place of rational demonstration," and that, in the present day of scientific inquiry, "the method of reasoning from established fact and fair induction, has effected much towards banishing the unnatural chimeras of imagination ; and it is hoped that the light of truth will eventually be enabled to penetrate into the more secret, and hitherto unexplored, recesses of nature," he only adds one to the multitudes of examples of the facility with which men can talk in the common-place phrases of the day about the errors of others and the true paths of knowledge, whilst they show that they really know nothing about these matters.

Dr. Power, before he advances his own, notices, according to the custom on such occasions, some of the hypotheses of the *efficient* causes of menstruation that had been previously advanced: he thinks the doctrine of its originating from "a peculiar *ferment* in the uterus," is "vague and unintelligible;" he uses, as if they were his own, the arguments of HALLER against the dependance of this function on "lunar attraction;" and he takes a similar notice of the conjectures respecting its "*final causes*," in which he, however, makes no mention of the most curious of the whole,—that it was intended to enable damsels and young widows to live decently, as we say, in spite of the restraints of our social institutes; an opinion seriously expressed in a Lecture to the College of Surgeons of London, in the nineteenth century!

Dr. Power opens his views of this subject with stating that "it [menstruation] has been regarded as the *sine qua non* of pregnancy; and it has been advanced, that women who do not menstruate, or *are not in a state disposed to menstruate*, do not conceive."—"Its connexion," he adds, "with the generative process cannot be denied; but it appears not sufficiently proved that its occurrence is necessary to enable the female to conceive;" and, in the next page but one, he quotes cases of pregnancy happening in women who had never menstruated ; so that, in this instance at least, he is pretty safe in his conjecture: and then, rising a little in his flight, he thinks that an "*improvement*" might be made upon the axiom, that "women who do not menstruate do not conceive," by substituting the following—"a woman menstruates because she does *not* conceive;" and of this he constitutes a principle which is the spirit of his hypothesis. Dr. Power thinks that, notwithstanding the manner the generative functions are influenced* by our social institutes, "the human female is so constituted as to be susceptible of impregnation, from earliest puberty, as various instances have demonstrated; nay, even before the establishment of the menstrual process. Under such circumstances, the occurrence of the discharge would naturally and necessarily be postponed during the nine months of utero-gestation, and, subsequently, through the period of lactation. As, at the latter part of this maternal function, it is common for the female to conceive again, previous to the re-appearance of menstruation, what is to prevent this repetition of pregnancy, and want of menstruation, throughout the whole period of the capacity for propagation? In this manner a female might go on through a long life, in the fullest discharge of the generative functions, without once menstruating. Nor is this an imaginary case: women have been known to proceed as above described; of which, cases may be adduced from respectable authority."—"Can it then be said," he asks, "that menstruation is a *necessary action*?" and here the negative inference he intends we should draw is, very aptly, supported by the fact of pregnancy having occurred without it; so that he argues correctly, if not very profoundly. These inferences, however, only lead us through the passages to his new and brilliant views: he proceeds to remark that,

"Were women living in a state of nature, is there not reason to infer that this discharge might be unknown? For, supposing (as that state would operate,) that, between the age of 15 and 45, the female was employed in the constant task of renewing her species, one nine months would be employed in producing, and the next nine months in nourishing, her child; so that no time would be left for its occurrence.

"It is now proposed to show, from physiological deduction, that menstruation ought not to be regarded as an absolutely necessary action in human generation; and to make an attempt to point out its real nature."

The author, after this, advances the following propositions:

"1st. That, in every female arrived at her peculiar age of puberty, and in whom the sexual organization is perfect, the generative process proceeds, in a regular and uniform series of progressive actions; to produce an ovum, containing whatever principle the female may contribute to the life and structure of the new creature, with nutritive matter to support its vitality and growth, until it is enabled to derive

* He says that, amongst us, "the occurrence of pregnancy, by the perversion of custom, is rather looked upon as an *accidental* than a *natural* state; so abortive are the generative actions liable to be rendered from such sources," (that is to say, "the operations of habit, education, moral restraint, and the customs of society.")

sustentation from sources external to that ovum; and also to prepare for its removal to, and establishment in, its appointed nidus.

"2dly. That this production of the female part of the embryo, and the preparation for its support and subsequent evolution, is entirely independent of all influence from the male."

These, too, are points which are not likely to be disputed. He then states that, "at and subsequent to the time of puberty, the enlarged ovaria are found to contain ova in different states of perfection; and, in women who have never been impregnated, corpora lutea and cavities, which have been supposed previously to have contained ova, have been detected: whence it may be inferred, that in them not only the formation, but the extrusion, of ova is accomplished, without the influence of the male." The last inference is probable; and, on these grounds, the author attempts "to show that menstruation is a farther link in the chain of these progressive actions, when they fail to be determined to conception by the operation of male influence;" or, as he has in another place more formally expressed it, that menstruation is "an imperfect or disappointed action of the uterus in the formation of the membrane, (decidua,) which is requisite for its connexion with the impregnated ovum."

We shall pass in review the arguments which the author has brought forward in support of this conclusion. He begins by asking, "whether it is likely that the uterine actions of the mature female should be confined to the ovaria, and consequent production of ova?"—"This is not only improbable," he replies, "but direct evidence may be advanced to the contrary, as in the sympathetic changes of the mammæ and the general system at the period of puberty, and the increased actions and propensities of the whole of the sexual organs. The uterus, in particular, enlarges, its vascular action increases, and the phenomenon of menstruation succeeds."

All this we must grant; it is a simple statement of what every one must have observed: but what are the purposes to which the author applies them?—"to show that the actions of the uterus, at the period of puberty, are influenced by the state of the ovaria." Such, Dr. Power remarks, was the opinion of CULLEN; but, as we should be guided by reasoning, not the mere authority of any man, let us adduce the arguments of this pathologist for his inference. Dr. Cullen says, (*First Lines*, 1001,) "As a certain state of the ovaria in females prepares and disposes them to the exercise of venery about the very period at which the menses appear, it is to be presumed that the state of the ovaria, and that of the uterine vessels, are in some measure connected together; and, as generally symptoms of a change in the former appear before those of the latter, it may be inferred that the state of the ovaria has a great share in exciting the actions of the uterine vessels." This, too, is a probable supposition; but there is such a prodigious chasm between these data and Dr. Power's conclusion, that we in vain endeavour to connect them, or discern the route by which the author has arrived at it. Immediately subsequent to the passage last noticed, Dr. Power says,

"It must be a matter of general observation, that the symptoms of puberty precede the menstrual discharge. The connexion of menstruation with the generative faculty is universally admitted, but the precise nature of that connexion has not been ascertained. It is conceived that the discharge is an effect of the actions of the uterus preparatory to its reception of the matured ovum, and which are disappointed in consequence of the stimulus of impregnation not being applied."

Nothing can be more incoherent than such reasoning, if this term may be applied to a string of phrases the sense of which is so utterly devoid of connexion. The author then fancies that he can discern in the human uterus, (assuming, at the same time, the point that menstruation occurs only in woman,) a mode of organization "which determines it to take on so peculiar an action:" but, though we admit that there is a peculiar structure in the human uterus, and that such a structure is adapted for the menstrual secretion, what support can this admission give to the notion that menstruation is an affection of such a structure, dependent on the *disappointment of the uterus in consequence of the stimulus of impregnation not having been applied?*" There is no connexion between the one and the other inference. Dr. Power, however, adduces here the formation of a membrane in the uterus in certain morbid states of menstruation, as a connecting medium: he compares this with that which occurs when impregnation is effected; he considers them to be of the same nature, and, consequently, to have a similar origin; and hence he infers a similarity in the causes of the two. This reasoning is very easily refutable. The membrane in question is not formed in ordinary menstruation; it is produced only in *morbid* states, when symptoms of irritation of the uterus are present; it is merely a membrane similar to that which is formed on almost all the surfaces of the body when a certain degree of what is called irritation is present; and it is an unwarrantable inference, that (even supposing that the membranes found after impregnation and during certain states of menstruation are identical in nature,) the causes of the two are the same, when we know that such is a common effect of various irritative causes affecting other parts of the body. We shall not dwell on the absurdity of the author's hypothesis; it is sufficient for us to show the badness of the reasoning on which it is founded: yet, as we wish to lay it before our readers in the most favourable manner, we shall adduce his summary of his doctrine of the consequences of the *disappointment* of the uterus in its expected visitor,—that is, the impregnated ovum.

"In proportion as an ovum acquires a state of maturity in the ovarium," Dr. Power says, "the uterus undergoes a correspondent preparation for its reception. Its vascular action is increased in a degree tending to a state of inflammation, and it wants but the additional stimulus of impregnation to determine it to the production of the deciduous secretion, so requisite for its proper connexion with the ovum: if, however, the stimulus of impregnation is denied, this increased action is not carried to a sufficient height to produce properly that effect; nevertheless, it is sufficient to give rise to the effusion of a fluid, which fluid is the menstrual fluid. The secreting orifices of the uterine vessels being thus determined to an evacuation of their contents, this proceeds until, the ovarian action ceasing, the irritations of the uterine system are relieved, and the effects of the increased local action obviated.

"The quantity of fluid discharged during menstruation, and the difference in the qualities of that fluid, from the decidua membrane, are not to be regarded as objections to this theory.

"The open mouths of arterial vessels, it is well known, will continue their effusion until the increased *impetus e tergo*, by which they are influenced, ceases,—as is evidenced in catarrh, gonorrhœa, &c.; and this will be more particularly the case where the discharge does not partake of a coagulating nature, as is the case in menstruation.

"Upon this principle, the uterus, being determined to the effusion of the menstrual fluid by its correspondence with ovarian action, continues that effusion during the continuance of that increased action. As this diminishes, whether in consequence of the relief given by the evacuation, or from the individual mature ovum having lost its influence by the disappointment of its impregnation, or both conjoined, the actions of the uterus diminish with it: menstruation then ceases.

"The duration of this period, and the quantity of discharge, will be necessarily determined by the degree of activity of the individual uterine system, as influenced by habit, constitution, or accidental circumstances.

"Nor is the colour of the menstrual fluid to be regarded as an objection to its congeniality with the deciduous membrane. Why the colour of the fluid should resemble that of blood, or why it possesses any other peculiar characteristics, it is as impossible and as unnecessary to explain as the peculiarities of any other secretion. It may be considered as most essentially differing from the deciduous secretion, in wanting the coagulating principle; probably from the uterus, under a minor state of action, not assuming sufficiently the inflammatory diathesis, or an approach to it, to produce the effusion of coagulable lymph. The difference of the menstrual and deciduous production is not more remarkable than the varied appearances which other secreting structures evince, under various modifications of action: increase the impetus of action, and the quantity of secretion is increased; carry this on to inflammation, and coagulable lymph is poured out, or pus formed. On the contrary, diminish the impetus, and the secretion is diminished, and becomes more thin, or serous; relax them, or diminish their tonicity, and they will even pour out red particles."

We stop here to notice the grossness of the author's views in physiology. Who else, in the present day, will think that such diversities in secretions result from a greater or less degree of *impetus of action?*

Dr. Power, in continuation from the paragraph last transcribed, proceeds to remark that the formation of the membranes, above noticed, in menstruation, is "a decisive proof that the generative actions of the human female, like those of the pullet, are capable of being carried to a considerable extent without the male influence;" and he immediately asks, "Does not the above fact afford most powerful evidence of the congeniality of the menstrual and deciduous secretions, if not of their identity? It, indeed, seems scarcely possible to doubt or contradict the identity of this variety of menstruation with the decidua, when the various arguments in its favour are accumulated." We have already remarked that, were it proved that the two membranes are identical in their nature, we should not be warranted in inferring an identity of their causes.

The following paragraph presents arguments that amuse one with their triviality, though we want something more valid than the author's assertion to make us believe that the sympathetic effects of impregnation, and menstruation with the formation of the false membrane in the uterus, are similar; and, with respect to the action which attends the expulsion of the membrane from the uterus, we do not doubt that Dr. Power would recognise an identical action were he to thrust his glove, or many other bodies, into the uterus. The passage we have to transcribe is this:—

"The membranous discharge differs not in appearance, structure, or in the sources from whence it is derived. It is attended by a series of sympathetic actions throughout the whole system, similar to what accompany that period of pregnancy when the true decidua is formed; it is also expelled by an action exactly the same as characterizes early abortion, so as to be even mistaken for miscarriage; and only differs in the circumstance of the ovum not having experienced impregnation."

In addition to this, the author remarks, that "this state of membranous menstruation appears very correspondent with the production and extrusion of the unimpregnated egg of the pullet, and is probably the effect of a similar excess of action."

The author is aware that this opinion of the identity of the causes of the two effects "is discountenanced by the fact, that women who are subject to such peculiar menstruation seldom, if ever, become pregnant; whereas, admitting it to be the consequence of a high disposition to discharge the generative function, conception ought to be more readily accomplished." He thinks, however, it is not difficult to reconcile the implied fact with his views; but his attempts to effect this consist only in suppositions. He says,

"The uterine actions are influenced by those of the ovaria: in order to a proper discharge of the functions of the former, it is requisite that a due and relative correspondence exist between these two parts of the generative system,—that, when the ovum is *mature*, the uterus should be properly *prepared* for its reception. Any derangement in the equilibrium of this relation will tend to derange the subsequent steps of the process: thus, if the ovum is matured before the uterus is prepared for it, the conception will be rendered abortive; *et vice versa*, the same effect will ensue if the uterus is too early prepared for the reception of the ovum. In these cases of peculiar menstruation, it is conceived, therefore, that an excessive local action of the uterus hurries it prematurely through its actions, while the state of progress of the ovum continues relatively in a minor degree, and incompatible with the correct generative actions.

"It may also be conceived, that the excessive action of the uterus, analogous with a state of inflammation, extending itself to the fallopian tubes, may render them impervious to the prolific influence of the seminal fluid, and thus impede their proper functions; or that the effused inspissated fluid may obstruct the communication through the body of the uterus itself."

His attempt to explain the periodical occurrence of menstruation in conformity with his peculiar notions, is, also, constituted of suppositions of the most gratuitous sort; and, as they do not occupy much space, we shall transcribe them, for the satisfaction of the curious and courteous reader.

"The generative powers of the human female are not limited to the production of a single ovum; on the contrary, a number may always be detected in the ovaria, under different states of progress. The loss or disappointment of one matured ovum is followed by the maturation of another: this, in its turn, becomes disappointed; and thus an indefinite series is carried on throughout the period of generative capacity.

"The interval between the maturity of two successive ova will be the interval between two successive periods of menstruation; and, as this usually occupies a lunar month, it is reasonable to infer that such time is required for the preparation or maturity of a second ovum, after the disappointment of a preceding one.

"In the human female, it is worthy of notice that, the period of puberty once arrived, the generative function continues uninterrupted, until, in the decline of life, it wholly ceases; while in other animals, and in vegetables, the powers of reproduction are only developed at certain periods; the sexual system, in the intervals, reverting to a state of immaturity. It is, possibly, in consequence of this continued orgasm, or excitability of the human generative system, that nature had deemed it necessary to relieve the periodical excessive actions, by providing the evacuation of the menstrual discharge."

"With regard to the *final cause*" (of menstruation,) the author says,

"It may be conceived that, looking upon the discharge as a deviation from what are to be considered the natural actions of the female, nature could have no final end in view, as it would be absurd to suppose an end contemplated where no natural efficient cause was provided. Nevertheless, some anatomical evidence may be adduced, which proves that the efficient cause, whether natural or unnatural, was foreseen, if not intended. How otherwise is the perforated hymen to be accounted for? the aperture of which appears evidently calculated for the transmission of the menstrual fluid.

"The hymen itself, which is peculiar to the human female, has, without doubt, some relation to her moral state, and may be considered as an evidence that the wise Creator intended to place restraints on the sexual passions of the human race, and their consequences, and is a fine illustration of the difference of its relations, with its beneficent Author, from those of the inferior animals. Our moral and religious duties are not unfrequently at variance with the dictates of nature; and it is to this seeming contradiction that the present paradox must be referred."

The arguments Dr. Power draws from the presence of the hymen respecting a phenomenon which, according to his views, should not naturally exist, and the other very curious inferences he founds on the same basis, happen, very unfortunately for them, to be utterly refuted by the fact that the hymen is present in several of the inferior animals. Haller, as well as our author, thought that this membrane was peculiar to woman, and that its presence indicated a moral law, the notion of which one may be sure originated with men and not with women themselves, like many other of our tyrannical regulations relative to this sex: but a membrane similar to the hymen is present in the female elephant, in several phocæ, the ape tribe; and CUVIER acknowledges, indeed, that DUVERNOY proved its existence in the females of all the mammalia. It arises, according to the ingenious view of Dr. VIREY, from a partial approximation of the raphé which runs down the middle of all vertebrous animals; and the manner in which he has elucidated this notion by his comparisons of it with the frena of the tongue and glans penis, and the structure present in certain cases of connate malformation of the genitals in men and women, renders his view highly probable. The fact of the presence of a membrane similar to the hymen in woman in several of the mammalia is, however, all we have to consider here, and it is indisputable. *Eh bien ! tant pis pour le fait*, was the only reply of a Frenchman to a fact presenting an obstinate objection to a brilliant hypothesis which charmed him: *tant pis*, then, Dr. Power may exclaim, for the facts in objection to his curious moral and theological reasonings.

Dr. Power's essay on "a Species of Absorption," presents, if possible, still more gross deviations from common sense or right reasoning than that which we have already noticed. He states in the title-page that he gives an account of a species of abortion, "not heretofore described," though no systematic writer on midwifery, for a century at least past, has neglected to treat of it: the merest tyro in medical study is familiar with the facts of abortion occurring in certain women at a similar period, in several successive pregnancies, and of the particular symptoms by which this accident is generally preceded. What has "not heretofore" been made known, is such an attempt at an explanation of it as is presented in this essay. Having expressed his opinion that abortion in such cases results from the fetus having become dead in the uterus, he endeavours to explain the cause of its death, as the prevention of this condition, he considers, presents the indication that we should conform to in our endeavours to prevent the remote consequences. He, in the next place, states our ignorance of the precise influence of respiration on the blood or system generally; but, he says,

"Whatever may be the real action induced, it appears certain that two important effects result. 1st. That an evolution of caloric is produced, affording the necessary stimulus of heat. 2d. That certain changes are produced in the electric properties of the blood, which adapt it to maintain the principle of vitality."

We interrupt our citation, just to remark that the latter inference is merely a vague conjecture; but precision on such points is of little import in the mind of Dr. Power: he, in continuation from the last paragraph, says,

"It is, however, by no means of importance which of the above explanations we may be disposed to admit with respect to the present point, as, *mutatus nominibus*, any will sufficiently answer our purpose; but, as it will be desirable to assume definite principles, in order to carry on a consistent argument, it is proposed to regard the actions of the respiratory organs as imparting oxygenous principles directly to the blood, and thus effecting the requisite changes in its constitution."

Let us, then, allow the author to assume a gratuitous term as the basis of his reasonings, and admit, also, the supposition that the death of the fetus results from a deficiency of this *unknown* principle acquired in the lungs of the mother, (in doing which, however, we have but little encouragement from facts,) and we embrace an hypothesis which might excite us to institute researches for the discovery of the nature of this unknown principle, not one that should make us assume our gratuitous supposition as a fact, and adopt practical measures accordingly. This, however, is what Dr. Power has done. He, at first, asks permission to use the *want of due oxygen in the blood* as a convenient term for an unknown condition; and then, as if this term was the expression of a known fact, proposes the respiration of an increased quantity of oxygen by the mother as a means of preventing the implied consequences of such a deficiency. This, with the administration of nitric acid internally, constitute the mode of treatment which is alluded to in the title-page, as "having secured a happy termination of the pregnancy, where previously repeated disappointment had been experienced." This is a striking assertion in the title-page of a book; but it would have been more consonant with professional decorum to have refrained from such a statement, unless some more valid evidence for it had been adduced than the two cases of which Dr. Power here gives the histories.

We are not willing to extend our censure of this book. We respect highly every attempt to improve the art of medicine by the advancement of physiology and pathology, and believe that Dr. Power has, in the publication of these essays, been influenced by the best motives: we shall, therefore, conclude with advising him to be in future less hasty in the promulgation of his thoughts; for a little deliberate consideration must enable him to discern himself his abuse of reasoning on this occasion, and must convince him that such crude and vague disquisitions will not favour his good reputation as a medical practitioner.

---

*Observations on some of the General Principles, and on the particular Nature and Treatment, of the different Species of Inflammation; being, with Additions, the Substance of an Essay to which the Jacksonian Prize, for the Year* 1818, *was adjudged by the Royal College of Surgeons.* By J. H. James, Surgeon to the Devon and Exeter Hospital, and Consulting Surgeon to the Exeter Dispensary. 8vo. pp. 328. T. and G. Underwood, London. 1821.

[In continuation from page 240.]

The next chapter of this work treats of the accordance of the general with the local affection. This accordance of disorder the author supposes to consist both in kind and in degree. This subject is illustrated by the manner in which the general ailment is increased or diminished in an equal ratio with the extent of the local affection, and altered in its character according to any change induced in the original seat of disease,—as by laying open foul abscesses, exposing sinuses, amputating mortified limbs, &c. He adds,

"If the secretions cease in a part from its being inflamed, those of the whole system are arrested or checked. When they recommence in the part, they are restored throughout the body, whether it be pus now formed, or the natural secretions: if, by treatment, the period when the secretions take place from the part should be protracted, as sometimes happens, the return of the general secretions is also delayed; while, if we can accelerate the one, we hasten the other likewise. If the local secretions are profuse and unhealthy, so generally are those of the system; and in this way, perhaps, the colliquative sweats and diarrhœa, which occur under profuse discharges, may be explained."

Now, although we are by no means disposed to agree with this explanation, which is simple enough; and inasmuch as the steps of the process are not very remotely traced, nor the connexion of the phenomena clearly shown, it is not very profound; yet it is something like an attempt at giving play to the reasoning powers, and at exercising the ingenuity.

The concluding sentences, although correct as to the actual accordance of diseased action in the system with the local disorder, shows more clearly the defect of this interpretation of their connexion, and at the same time points out the necessity of referring them to another cause, as the simultaneous effects of its influence.

There is no doubt that any noxious influence may primarily affect any one part of the body in such a manner as, by means of the usual channels of impressions, or through the medium of the circulation, may give rise to a similar, or at least corresponding, disorder throughout the system; but care ought to be taken, in our interpretation of the progress of phenomena, to distinguish between those which are produced after this manner, from those which commence in the system from constitutional and other causes, and which may appear in any particular organ and texture, co-ordinately with, or in consequence of, the general derangement.

We shall continue our quotations, exhibiting the author's opinions on sympathy.

"If mortification occur, from accident, in an individual previously healthy, in a few hours he shall be reduced to the same state as another in whom this affection originated from long error in health.

"A man shall have compound fracture, and in the advanced stages of that calamity bleeding would sink him: let a vital organ inflame from cold or any other cause, and the same depletion, which a few hours before would have destroyed him, now will save his life."

After making some general observations on sympathetic fever, for an account of which we must refer to the work, the author continues:

"As the constitutional affection is influenced by the local disease or injury, so is the latter by the former, and we may suppose *simili modo*; but here we have access to fewer facts. One of the most unexceptionable proofs, however, is to be found in the history of amputation: for, if that be performed after injury, previous to the accession of fever, it generally does well; whereas, if afterwards, the state of the constitution becomes the cause of the stump inflaming, and often doing otherwise."

This is certainly the correct state of the case, and places the much-controverted point of the best period in which to perform this operation in a perspicuous view.

Mr. James makes some very just observations on the causes which influence the progress of inflammation, by affecting the system generally. Respecting these, he first notices the state of the prima via, and disordered action in the liver: to the latter he appears, however, to attach too great importance. The quantity and quality of the blood are next considered, and the effects of the former very properly appreciated; but in too general terms, and without inquiring either into the origin of such an impure state of the circulating fluid, or its mode of operation on the animal economy. In justice to our author, we must remark that, to have entered upon this subject with the degree of fullness to which it is certainly entitled, or as it respects even the production or the modifications of inflammation, might have led him into a disquisition too much at length to accord with the brief, yet extended, view he has exhibited of the various kinds of inflammatory action. We shall give all he has said upon this subject.

"An impure state of the blood exists, perhaps, more frequently than we are aware of; but, as it is invariably connected with disorder of the digestive organs, the effects which partly arise from both causes are often exclusively attributed to one. But, when I see persons in whom every scratch festers into an ulcer, as in scurvy or scrofula;—when I observe that the atmosphere alone will change the disposition of every action;—that poisons introduced, and acting upon the circulating medium, will induce the most powerful effects upon the whole system;—I must profess myself to be a humoralist in a considerable degree, (although quite ready to recognize the direct, as well as the indirect, influence of the digestive organs and nervous system on disease,) a doctrine which Mr. Abernethy has himself most perspicuously enforced, with reference to many; for, although he prefers the general explanation of the phenomena of disease by sympathy, yet he does by no means exclude the influence of a depraved state of the blood.

"If experimental research often instructs, I believe it also frequently deceives us, because we too hastily form deductions from what we deem conclusive experiments. Now, the opposition to the humoral pathology has chiefly been grounded on the fact, that we cannot discover such an alteration in the circulating fluids as would seem to us to justify the supposition that it exists. When, however, we see the efforts and the expedition with which the animal economy rids itself of some foreign matters, which we can recognize by their specific effects, as ipecacuanha or jalap introduced into the veins,—or by their sensible qualities, as mercury, nitre, asparagus, turpentine, garlic, &c. introduced any how,—we must be strongly prejudiced not to believe that those matters have been contained in the blood, even although in the blood we cannot perceive them. But, do not the vital powers quell and suspend the chemical properties of most substances? Do they not convert the most dissimilar matters into an apparently similar substance? May they not prevent these, and others, from manifesting their specific qualities, which nevertheless, though modified, are not completely subdued, and may still excite great disturbance in the constitution? And, if so, may not those powers, with reason, be supposed to prevent the sensible alterations in the qualities of the blood, in such diseases as scurvy, scrofula, gout, lues, mercurial disease? And, if we are compelled to admit such alterations in these, may we not be called upon to allow that it possibly exists in a greater or less degree in many others? The abuse of the humoral pathology has led to infinite mischief; but, if we are content to recognize the truth of the doctrine as far as facts seem to establish it, without however grounding any theory or practice on that which we only imperfectly understand, no harm can arise from belief. We can never err while we consider experience paramount."

Of the importance of the constitution of the blood, in giving rise to, or modifying, and even in removing, the phenomena of disease, we have been long and very fully impressed, and we hope soon to bring the subject more fully before our readers; but we do not take that strictly humoral view of the subject with which the author is in some degree impressed. Nor, in our opinion, does scrofula, nor indeed any other hereditary disease, owe its origin to an impure state of the circulating mass of fluid: it is in other diseases where we must look for the effects of such a course. While we accord with the principle, we would differ from him in the mode of illustrating it. As to the opinion expressed in the form of a corrollary, " We can never err while we consider experience paramount," it must, in the science of medicine at least, be considered, if implicitly followed, as exerting an unfriendly influence, and proving of difficult application, if directed with the true philosophic exactness which ought to characterize our researches. It is already too much the spirit of the age to worship Experience as the enthroned divinity, while she is only the elder priestess ministering in the temple of medical science.

After adverting to a diseased state of the vessels, he nex glances at the influence of air and temperature.

"The influence of air and temperature seems to be immediately connected with the state of the circulating fluid, on which their agency is primarily and principally exerted. Their influence is recognized, but not, perhaps, generally attended to, in the full extent it requires. We know that the same disease goes on very differently, if the patient be situated at the top of a hill, or in a low valley,—or on an open heath, or in a jungle or marsh overspread with pasture and foliage;—in the open country, or in a populous town;—in a spacious, clean, and well-ventilated apartment, or in a small, close, and dirty room;—nay, in the upper story or ground floor of the same building; or even if well elevated above the floor, or placed upon it. I believe there is no poison more injurious than foul, no restorative more effectual than pure, air; and it runs no risk of disordering the digestive organs, as bark often does, or stimulating the vessels too much, like wine. It is always safe and always useful.

"If it be demanded, upon what system the air chiefly acts? I should again say the blood; for, if we take a man in previous good health, and place him in an hospital, for an injury not very severe, at a time when the cases there are doing badly, and find that, without any cause which can particularly affect his alimentary or nervous system, a bad kind of inflammation is set up, is it an unreasonable conjecture that the blood which, in the lungs, is particularly exposed to the contact of this air, should be thereby rendered impure?"

The proposition which this sentence contains,—namely, that respecting the direct influence of the impure air upon the blood in the lungs, and which excluded any particular effect "upon the alimentary and nervous systems," is apparently contradicted by the concluding part of the same paragraph:

"It must be understood," the author adds, "however, that although blood rendered impure, by this or any cause, is likely to afford but a bad material for the processes going on in an inflamed part, yet that its operation on the nervous system generally, and through this on the alimentary, is perhaps equally, if not more, injurious."

Now, here we must accuse the author of a want of precision in the order of phenomena which he describes, and also of the terms which he uses. Hence arises an apparent contradiction of his opinions in different portions of the same paragraph.

The influence of air in the production and removal of disease, is evident to every one; but, the exact process it pursues in producing these effects may be a subject of dispute.

We by no means coincide with the author in the supposition that vitiated air produces its primary effects upon the blood. Such will not always be the case; and, when it does act in that manner, the effect will arise from its impeding the decarbonization of the blood, and not from any actual impurity that may be communicated to that fluid.

The influence produced upon the circulation will be even opposite, or at least various according to the nature of the impurities existing in the atmosphere; but we have pretty good reason for supposing that they produce their effects upon the circulating mass in two ways, either separately or in combination, according to the nature of the impurity upon which their effects depend; and that in no instance can it be shown that any impure gas is communicated to the blood circulating in the lungs during respiration, while there is every reason for supposing that they act in a much more circuitous and indirect manner; the consideration of which neither the opportunity nor our limits permit us to enter upon.

Respecting the influence of *temperature*, the author thus expresses himself:

"With regard to temperature, the history of the diseases of different climates very clearly shows that it exerts a most important influence; yet it may be much doubted whether this is owing to the direct agency of heat. It should rather seem to proceed from the changes it induces on the atmosphere, by its action on vegetable and animal matter. Many mechanics, as anchor-smiths, glass-blowers, &c. almost live in a temperature far higher than the tropics; but, although they are but too apt to indulge in every species of excess, yet their diseases in no degree resemble those to which the inhabitants of tropical climates are prone. Under the same temperature, different districts are far more unhealthy than others, and their diseases are marked by peculiar characters. The diseases of spring and autumn, when the temperature is the same, are very different. This has been accounted for by supposing that the heat of summer has occasioned a certain alteration in the system, and to a certain degree this is the case, no doubt; but, are persons coming to this country, in the spring or summer, from warm climates, subject to autumnal diseases? I believe not; and they should be if this were the only cause. Cold certainly occasions a strong disposition to inflammatory diseases."

However much we may be obliged to the author for considering the influence of temperature upon inflammations, yet we think this notice too brief when we view the importance of the subject. In one point we agree with him,—namely, in the sentence where he considers the effects of temperature as chiefly resulting from the changes it induces on the atmosphere, by its action on vegetable and animal matter. This is certainly the case to a considerable extent; but we are still convinced that it produces considerable influence upon the system in a more direct manner. The reasoning of the author in support of his opinion is both desultory and illogical. The temperature to which an anchor-smith is exposed is no more akin to an intertropical temperature, than the radiant caloric of the fire of his forge is similar in constitution to the solar beams. We are even tolerably convinced that the powerful rays of the sun influence the human system in a different manner from what might be expected to result from an equal quantity of uncombined caloric: as the solar beams are compound, so we may expect them to produce a more complicated effect upon organized matter. But, even granting the operation of both species of temperature to be the same, (which is certainly not the case,) the mechanic does not pass his nights under such an influence, at a time when the system is more liable to suffer from its operation; nor, when labouring under disease, is he subjected to such a source, not only of its primary induction, but also of its continued aggravation.

The author pursues the subject of the causes which influence the progress of inflammation, by considering the state of the nervous system. The manner in which this part of the subject is treated accords with the usually-received opinions upon the subject, but not altogether with our own. Our space cannot permit us to oppugn them, had we even the inclination. Under this head, he also considers the modifying effects of strength and debility.

The following chapter treats "on the purposes and uses of the different modes of inflammation." It is written a good deal in the spirit of John Hunter, and contains some very good remarks, although we could find fault with several of the doctrines there taken for granted. The various states of the parts in the different kinds of disease, and in their separate stages, are well described and remarked upon: but the process which nature pursues in effecting these changes are not traced; and, when the attempt is made, agencies which we cannot admit to exist already formed in every texture, are too often brought in aid. Nor are such agencies themselves merely passive effects of more remote causes; they have too frequently a surprising degree of intelligence assigned them, and which, being at best but a supposition, we cannot admit of entering into the explanation of pathological views. We could multiply examples, but the following, taken at random, affords rather a curious specimen of reasoning from the effect to the cause, and the uses to which this local intelligence, in the plenitude of sagacity, converts both.

"Mortification also has its use; for, supposing a part is so injured that it can never return to a healthy state and action, it would prove a perpetual source of irritation to the body during the remainder of existence; but the economy possesses a power of killing it, and then the ulcerative process separates it. If a poison be applied, a slough is formed, as in some cases of pressure, to cut off the communication between the injurious cause and other parts, and thus protect them from its operation."

We are much of opinion with Hobbes, that such reasoning is "entitled to the privilege of absurdity;" and, were we inclined to be poetical, we would be disposed to exclaim, with Cowper,

> "Such reas'ning falls like an inverted cone,
> Wanting its proper base to stand upon."

This intelligent guardian is certainly not argus-eyed, since he frequently permits the invasion of the enemy without resorting to these efficient means of defence.

This chapter of the work ("on the purposes and uses of inflammation,") notwithstanding the absurd title and the frequently ridiculous doctrines which it implies, yet exhibits a comprehensive detail of the phenomena or stages of the disease which the author has thought proper to view in that light. We, however, think that his essay would have assumed a more connected and scientific appearance if, instead of considering this part of the doctrine of inflammation in the character of an intended purpose, expressly entered upon by the animal economy, in order to fulfil certain limited offices in the system, (which is the view of the subject taken by those of the same school,) he had considered it in connexion with the previous phenomena, and as the consequence of these, and the modifying causes, producing varied, or even opposite, effects, according to the extent of their individual operations; which, in their turns, serve farther ends, and give rise to a more extended influence in the living body. There cannot be the least doubt but the effects that result from certain processes going on in the animal constitution become, in the regular procession of phenomena, the causes of farther effects, which, in uniformity with the grand laws of nature, tend not to one, nor even to a few, specific results only, but give rise to a varied influence, according to the multiplied causes which modify and direct their progress.

It would have been more consistent with the order and variety observed in the operations of the animal system, had the author viewed this particular part of his subject in connexion with the previous changes, and with those which, as an intermediate process, it is destined to produce, and which may terminate variously according to the number and intensity of the causes that may influence its progress. These intermediate stages of inflammation would have been sufficiently recognized in "*their purposes and uses*," by the manner they would have tended to give rise to specific effects, from the peculiar action which they might assume, and according as such action might produce any other, whether terminating in health or in that immediately preceding the death of the part disordered, or the dissolution of the whole system.

The author, in the next chapter, treats the subjects connected with *mortification* in a tolerably satisfactory manner. We cannot afford space for the examination of the pathological doctrines which it exhibits. The succinct manner in which the causes capable of influencing and accelerating the progress to this state of parts are enumerated, will be our excuse for giving it in his own words:

"There are many circumstances which are capable of influencing, in a very great degree, the disposition of inflammation to terminate in mortification, whatever may have been the original cause of that inflammation: this is not to be wondered at, since most of these are of themselves capable of inducing the state of mortification. When they do so, they deserve separate consideration; but, in every case of inflammation which occurs, where there may be any reason to fear this termination, it is necessary to bear in mind that their operation will much tend to influence its production; and many of them may be avoided by attention, or counteracted, perhaps, by proper means. They may be stated as follows:

"*a.* The powers of the part in which inflammation occurs being naturally weakened, as in fibrous membranes, or in the scrotum, &c.

"* *b.* The remote supply of blood or nervous energy, as in the lower extremities.

"* *c.* Obstruction to the return of blood, from depending position, pressure, or actual ligature.

"* *d.* Obstruction to the supply of blood, from pressure or ligature; rarely from the former, except in aneurisms.

"*e.* The circulating powers being impaired, from disease in the heart or vessels.

"* *f.* The powers of the constitution being enfeebled, from age, the influence of habits, disorder of the digestive organs, or actual disease, as fever.

"* *g.* An impoverished or diseased state of the blood, from poor living, foul air, improper food, scurvy, &c.

"*h.* The organization of the part being much impaired by external injury.

"* *i.* The nervous power of the part being impaired by poisons, &c.

"* *k.* The actions of the part being unduly excited by any excessive irritation, particularly when its powers are weakened.

"* *l.* The powers of the part being unduly lessened by the unseasonable application of depressing remedies.

"* *m.* The part being either subjected to pressure from some foreign cause, or produced by the tension of skin or aponeurosis, &c. in consequence of the swelling.

"* *n.* The excessive violence of inflammatory action.

"*o.* A peculiar disposition in the constitution.

"Of these, many may either be avoided, diminished, or counteracted, and such are marked in the margin *; and, with respect to the remainder, we are not without means of limiting or checking their influence.

"The excessive violence of inflammatory action has been stated as a cause, but it is more frequently the consequence, of the action of some of the above causes. *There must be a reason for inflammation being excessive.*

"Under heads *c*, *k*, *l*, and *m*, will fall nearly the whole of our remedial means; and, upon the proper regulation of temperature, applications, medicine, diet, and depletion, according to the nature of the case, the success or failure of our efforts will depend."

The author now comes to the second part of his work, in which he treats of the different kinds of inflammation. This is done in a systematic and very succinct manner, after the arrangement which has been proposed by himself.

We deferred the consideration of the grounds upon which this classification is founded until now, (although the author discusses the subject in the earlier part of his book,) in order that our observations might accompany the arrangement itself, and both be in closer connexion with our analysis of the most important disorders which it embraces, and which, in the work before us, is briefly but correctly delineated. The author states that—

"The arrangement of inflammations here proposed is founded upon the following facts:

"1st. That the mode of repairing injury, and of arresting the progress of inflammatory diseases, depends upon the power which the animal economy possesses of effusing organizable lymph. If this exists in any given case in a sufficient degree, the progress of the inflammation will be *limited;* if the contrary, it will *spread*. And the danger of the disease being in proportion to the disposition to spread, *cæteris paribus*, more constitutional sympathy, denominated sympathetic fever, will be excited. And this sympathetic fever, however salutary in its nature, will, when it exceeds certain bounds, tend to augment rather than lessen the mischief.

"The disposition to spread may be owing either to the nature of the part, as a surface; of the cause, as a poison; or to the state of the constitution: but the former are circumstances more or less *accidental*, and, though very important, cannot afford a basis of distinction; but the latter, as a permanent cause, certainly will; and, when it is similar in nature and degree, and accompanied with the same concomitants, it will be found to produce the same effects, &c."

This disposition "to *limit* or to *spread*," the author assumes as the basis of his classes. In our opinion, such a disposition, even granting it to be a specific and inherent attribute of the constitution, according to Mr. James's mode of viewing the subject, yet it by no means constitutes a distinctive character of inflammatory disorders, down through their various distributions into genera and species. For the majority of such maladies may evince either of these properties, or both of them, at different periods of their progress, owing to the varying degrees of nervous energy, the state of the internal organs, or the influence of external causes. And we are inclined to think that, in many instances, especially at the commencement of such diseases, it will be no easy matter to determine the class to which they ought, with strict propriety, to be referred. This difficulty, therefore, as it originates in the constitutional causes of the disorder, must, notwithstanding the author's opinion of the contrary, exert an important influence upon our practice during their early stages.

The degree of connexion which the part diseased possesses with vital organs or functions of the animal, because of the influence thus exerted over the character of the inflammation, is assumed by the author as the basis of the distribution of his orders. In support of this part of his arrangement, he only adds, what requires illustration, that such influence "acts invariably, and, *cæteris paribus*, in the same degree; the constitutional sympathy being in proportion to the danger, the difficulty of resisting that danger, and of repairing the mischief done." The author proceeds as follows to characterize his genera.

"*Thirdly*. There is a circumstance in the history of inflammations which has hardly received a due share of attention, but it is both sufficiently remarkable and very important; namely, the original disposition to terminate in one mode rather than another: thus, in boil and whitlow, it is to suppurate; in carbuncle, to slough; and in mumps, to resolve; and this disposition is so strong, that it is very difficult to procure any other termination. It may happen, however, that there shall be more than one mode in which it is disposed to terminate,—as in either resolution or suppuration, in sphacelus or ulceration, and so on. On this principle I have grounded the establishment of the genera."

In order that our readers may form their own opinions upon the merits of the author's classification, we shall exhibit it at full length, as it is drawn up by himself, and appended to his book.

### NOSOLOGICAL TABLE OF INFLAMMATIONS.

### *Inflammations from External Causes.*

*Mechanical* injuries by incision }
contusion }
laceration }
of integuments or muscles
tendons or ligaments
bones and joints
membranes
parenchymatous structures.

*Mechanical* injuries from pressure
interruptions to the circulation by bandage
ligature.
*Chemical* injuries from excess of heat..............burns and scalds.
cold..............frost-bite, chilblains.
from poisons, such as strong acids, metallic salts, alkalies, poisonous reptiles and insects or mammalia, various vegetable matters.

### *Inflammations from Internal Causes.*

Originating from disorder of the constitution more or less peculiar in most species.

### CLASS FIRST.

Disposition to limit the inflammation by the effusion of organizable lymph.

*Order First.*

Affecting vital organs.

*Order Second.*

Affecting parts of great importance.
Inflammations in joints.
the eye.
the testicle.

*Order Third.*

Affecting common or external parts.

*Genus* I.—*Char.* Disposition to resolution or chronic induration.
*Species* 1st. (Acute,) Parotidæ.
2d. (Subacute,) Inflammations. *a*, in the mamma.
*b*, in the lymphatic glands (not scrofulous).
*Genus* II.—*Char.* Disposition to resolve or suppurate.
*Sub-genus, a.* In which there is considerable disposition to resolve.
Phlegmon of cellular membrane in general.
of lymphatic glands in general.
*Sub-genus, b.* In which the disposition to suppurate predominates.
*Species* 1st. Furunculus mitis.
2d. Paronychia mitis.
3d. Abscess in young persons, juxta anum (phyma).
juxta urethram.
*Genus* III.—*Char.* Disposition to unhealthy suppuration and mortification, but in some cases susceptible of resolution.
*Species* 1st. Furunculus gravis.
2d. Carbunculous abscesses.
*a.* Angina externa.
*b.* Abscess juxta anum } in advanced life.
*c.* urethram }
*d.* Anthrax on the penis, &c. &c.
3d. True carbuncle.
4th. Pestilential bubo.

### CLASS SECOND.

In which the inflammation spreads, from the disposition to limit being deficient.

*Order First.*

In vital organs, *e. g.*
In pyrexia of an asthenic nature, as typhus, puerperal fever, measles, scarlatina maligna, small-pox, dysentery, in aphthæ, &c.
In the vascular system.—1st. In absorbents.
2d. veins.
3d. arteries.

*Order Second.*

In external parts, or those of secondary consequence.

*Genus* I.—*Char.* Disposition to produce a temporary increase, and morbid state of the secretions of the part, and to resolve.

*Sub genus, a.* Superficial inflammations of the skin. (Cutaneous inflammation.)

*Sub-genus, b.* Catarrhal inflammations of mucous membranes.

*Genus* II.—*Char.* Disposition to unhealthy suppuration.

*Spec.* Inflammations of { 1st. Cysts of chronic abscesses / 2d. bursæ / 3d. tumors } when open.

*Genus* III.—*Char.* Disposition to resolve, or to suppurate and slough.

*Sub-genus* I.—Erysipelas.

*Species* 1st. Erraticum.

2d. Phlegmonodes.

*a.* Verum.

*b.* Biliosum.

Complicated with inflamed absorbents.

3d. Œdematodes.

*a.* Biliosum in old and debilitated persons.

(E. Gangrenosum of many authors.)

*b.* Dependent upon other diseases.

*α.* attending sphacelus from ossified vessels.

*β.* from sympathy, where there is deep-seated matter.

*γ.* conjoined with, or produced by, anasarca.

*δ.* from varicose veins.

*ε.* consequent on bruises.

4th. Infantilis.

*Sub-genus* II.—Paronychia gravis.

*Species* 1st. In thecâ tendinum.

2d. Sub-periosteo.

*Genus* IV.—*Char.* Acute. Disposition to phagedenic ulceration and sphacelus, produced by morbid poisons.

*Species* 1st. From venereal poison.

2d. mercurial.

3d. Gangrena nosocomialis.

4th. In the labia of female infants.

5th. Malignant pustule.

*Genus* V.—*Char.* Chronic. Disposition to mortification.

*Species* 1st. From pressure in unsound constitutions;—in old people; during fever;—during other diseases.

2d. From the effects of poverty, scurvy, &c.

3d. disease of vessels.

4th. . . . the use of unsound rye as food.

The author now enters upon the consideration of inflammations resulting from external causes, and treats first of those which arise from "mechanical injury." His observations on this occasion appear to be judicious, and are arranged in a systematic and lucid manner. The treatment recommended is well conceived and discriminative. The chapter on inflammations from chemical causes he subdivides into—1st, those from the effects of temperature; and, 2d, from poisons. The remarks on inflammations arising from the excess of temperature are not sufficiently copious, but those which are offered are correct. The recommendation "of opium and cordials in the first stage" of injuries of this nature is, however, given in too general terms, and in a manner insufficiently laudatory, when the admirable effects of these remedies, especially the former, are taken into consideration: nor, in our opinion, ought their employment to be restricted to the first stage; there are instances of such injuries which, owing to the peculiar circumstances of the case, require this treatment in their more advanced stages. The author, after noticing the delusive data afforded by the thermometer of the production of animal heat in a scalded surface, adds, that this instrument "will not at all ascertain the quantity of heat a part is giving off, or capable of giving off; for the thermometrical temperature of two parts shall be the same, the one being inflamed, and the other near the centre of the circulation; but the quantity of fluid which the first will evaporate shall be two, three, or four, times greater than the other." He proceeds also to observe, that inflammation arising from scalds is not the only species of this disorder which is benefitted by applications of an opposite nature,—by heat and stimuli, and by cold and sedatives. After offering farther observations upon this kind of inflammation, Mr. James discusses that which results from *cold* in a few words: we shall select the passages worth the trouble of transcription.

"Perhaps the principle on which inflammation is produced by the application of cold, is not so entirely different from that occasioned by the direct influence of heat as might be at first supposed.

"Cold does not seem to produce inflammation *per se:* a person or a part may be kept in a great degree of cold, and no inflammation will occur; but inflammation takes place when heat is applied to a cold part; and that is heat, to all intents and purposes, to a part which has been exposed to a temperature many degrees below the freezing point, which to others is temperate, or even cool; and while, on the one hand, such a part is liable to be very injuriously affected by a slight degree of warmth, so, its powers being much diminished, it is less capable of resisting an injurious impression of any kind, from the same reason that a slight degree of heat will produce vesications in a limb whose powers have been reduced by paralysis.

"Observing, then, that parts exposed to a violent heat inflame, if afterwards subjected to a mean temperature, but do not if gradually cooled;—observing, also, that parts which have suffered extreme cold also run into inflammation if subsequently exposed to a mean temperature, but do not if gradually warmed; we may infer that the inflammation in either case is less the consequence of the immediate application of heat or cold, than of the sudden change which takes place: nevertheless, I do not mean to deny the direct influence of excessive heat or cold; for, when they are sufficiently intense to disorganize the parts by their action on the vessels, they induce mischief which cannot be remedied by any thing but inflammation."

The opinions just stated have been promulgated, as the author has shown, by LARREY and PEARSON, and are so far correct; but there still remains much to be done in farther elucidation of the pathology of this species of vascular lesion.

Respecting inflammations resulting from the application of *mineral* and *vegetable* substances, considered in a strictly surgical point of view, the observations of the author are necessarily brief. On the *animal* poisons he makes the following observations.

"They are of two kinds: the one acts violently and immediately on the part to which they are applied, exciting severe inflammation, but having nothing in common with the living properties of the animal, which therefore throw them off, if they are able to survive the first injurious impression; such are the poisons of reptiles, insects, &c. Their *modus operandi* would appear to be by the direct destruction of the vital principle of the part, or animal, on which they operate.

"The other excite less intense inflammation, have a certain relation with the vital properties of the animal, in whose constitution they produce a peculiar alteration; but are sooner or later eliminated from it, if they do not previously destroy life. Such are the poisons of other mammalia, particularly of man himself."

The treatment recommended locally, in the first class of animal poisons, is the same with that given by BOYER in his "Traité des Maladies Chirurgicales." Cordials, as ammonia, &c. and also arsenic, are to be exhibited internally.

We now arrive at that part of the work which treats of inflammations from *internal causes.* These the author believes to proceed "from some defect inherent in the part, or some error in the constitution," either hereditary or acquired. The constitutional indisposition may be permanent and peculiar, constituting a diathesis: under such circumstances, "any inflammation arising spontaneously will be impressed with peculiar characters," and will form peculiar kinds; as the gouty, the rheumatic, and the scrofulous. There may also exist other states of constitution, which, arising from peculiar causes, operate specific kinds of inflammation, and whose duration is various; as from measles, the venereal poison, &c. These result from evident and specific causes; but there are other states of the system, recognized with greater difficulty, unless in their effects, and which can seldom be clearly traced to an obvious cause; as phlegmon, or carbuncle, or erysipelas, &c.; "but, though peculiar, they are not permanently so, and the person who now has erysipelas may, in a short time, have phlegmon." After making some farther general observations connected with this subject, and upon the disposition which inflammation may assume to limit or to spread itself, the author proceeds to the consideration of such disorder in vital organs. These he ranks in two orders, according as "their functions may be more or less immediately connected with the support of life." In the first is arranged the stomach, small intestines, brain, and the heart. In the second rank is placed the lungs, liver, and kidneys; because, "although the interruption of these will sooner or later be followed by death, this consequence is not so immediate, nor are the attendant symptoms so urgent. This is still more the case with the pancreas, spleen, bladder, and uterus." To us this arrangement appears arbitrary enough; and we are at a loss to think why the brain ought to be considered an organ more essentially vital than the lungs. Instances of the phenomena of life being continued for some time in several of the more perfect animals, although not a vestige of brain could be seen, are on record: others may be furnished wherein this organ was found ossified throughout, and yet the animal remained in apparent health; and, in the lower part of the scale of animated nature, there are whole orders that may be adduced in proof that the existence of a brain is not even necessary to the continuance of life. The same cannot be said of the lungs: all animals have either such an organ or an apparatus which performs the same office in their economy. We consider, therefore, the lungs as a vital organ of the very first importance in all animals.

Inflammation affecting serous membranes is next noticed, and the disposition to diffuse itself in this texture is especially considered. The proneness of these membranes to the spreading inflammation, the author accounts for "from the circumstance of their being ordinarily exempt from all injurious impressions, and therefore may be less capable of resisting them when they do occur; and because all the results of inflammation, except resolution, must be productive of consequences injurious to the functions of the organs they envelop." We must confess ourselves but little wiser for this explanation, and as little from the following attempt to show why adhesion takes place, as a consequence of inflammatory action, more frequently than any other mode of termination.

"The disposition to adhesion certainly predominates, and that for reasons which Mr. Hunter has assigned; namely, because effusion of any kind of fluid into a shut cavity is injurious, and therefore, under inflammation, the natural disposition is to exclude this; and it rarely occurs, excepting under an uncontrollable degree or of a peculiar kind, as in some cases of dropsy."

To say that such an effect is not produced because it is injurious, is only one way of expressing our ignorance upon the subject, and conveys us not one step towards a solution of the difficulty. Had the author, instead of having his attention absorbed by the distinctions which he has moulded into a system, applied his observation more closely to those which are the result of the texture in which the inflammatory disorders may be situated, he might have more readily discovered a reason why inflammations seated in serous membranes more readily throw out coagulable lymph and form adhesions, than when they occur in any other tissue.

He next proceeds to offer some observations on mucous membranes: these are not viewed "as vital organs" by the author. We are disposed to differ from him in this. According to the common acceptation of the word organ, they may not certainly be considered as such; but, if the separate textures of the body be individually considered in their dependencies, and in the phenomena which they are engaged in producing, no tissue can have greater claims to be considered vital. In enumerating the kinds of inflammation which are observed in this texture, he says, "it may also be their original disposition to ulcerate and slough." We showed the fallacy of this in the former part of this review, and that such affections were the result of the inflammatory action having extended to the adjacent cellular tissue. The causes which produce inflammation in vital organs appear to be correctly enumerated, and therefore afford no grounds for remark. The indications of cure are very briefly, but judiciously, suggested. Of the treatment of wounds in vital organs, he speaks as follows:

"Where nature is herself capable of curing disease, we should do well to observe her processes, and to imitate them; for she is not to be thwarted, but obeyed. If a man receive a wound in a vital part, we observe that he drops in general, and there he would lay, perhaps, in a state of inaction, and under the influence of privation, if he were not removed. The wounded soldier does often remain in such a predicament, without help, but without the stimulus of warmth or food, and the processes which are immediately consequent on the wound are not defeated by these causes, and by being moved prematurely; while his comrades, carried to a crowded house or hospital, very frequently die. As syncope from hemorrhage, though alarming as a symptom, is beneficial as a process, so the sudden and entire loss of power which often follows a wound in a vital part, may contribute greatly to prevent mischief, by the contraction of vessels it occasions, and its incapacitating the individual from moving or procuring food."

The author proceeds next to the consideration of "limited inflammation of parts of great, but not of vital, importance," as of the joints, the eye, and of the testes; when he arrives at the third order of this class, namely, "inflammation affecting common or external parts." Having disposed of these in a very satisfactory manner, and in the order given in the appended table, the *second class*, or those which possess "the disposition to spread, from failure of the adhesive process," is afterwards brought under consideration. He introduces this subject by the following observation:—"It is not to be understood that I have any intention of asserting that the disposition to limit is entirely absent in this class of inflammation: on the contrary, there appears to be a struggle in the part and the general system, arising from the endeavour to effect that which they are either totally or for a long time incapable of performing." This, in our opinion, is a formidable difficulty against the classification recommended, and, as we have before suggested, almost insurmountable, whether viewed in its pathological or therapeutical relations; for, the treatment employed in the majority of inflammatory diseases, and other causes, may change their character in such a manner that the disorder, which to-day was disposed to limit itself, may to-morrow assume a directly opposite character, and *vice versa;* and thus it will alternate from one class to the other, without being long possessed of either characteristic. Mr. James considers, under the head of "spreading inflammation in vital organs," this action occurring in absorbents, veins, and arteries. On inflammation in veins, his remarks are more extended than usual. With respect to arteritis, he adds little, and appears unacquainted with what has been lately written upon this affection. After disposing of these subjects, he arrives at the second order under this class, namely, "inflammation, having a disposition to spread, situated in external parts." Under this head he arranges the majority of diseases of the skin and mucous membranes. On chronic abscesses, tumors, and bursal inflammations, which possess a disposition to an imperfect and unhealthy suppuration, the remarks are concise, but those which are offered are judicious.

The author enters more at length on the consideration of erysipelas, and in such a manner as to lead us to consider the portion thus occupied the best part of his book. We can confidently recommend it to the perusal of our readers, for the correct and concise views it exhibits of the semiology and treatment of the different varieties of this disease. He considers *erysipelas phlegmonodes biliosum* the most common form of the disease. After describing, in a very succinct but full manner, the causes, characters, and progress of the disorder, he enters upon the treatment, which he discusses satisfactorily. He recommends bleeding, when the pulse is full, strong, or bounding; when the skin is hot, and the part is much loaded; and "where the tension of the surface indicates a considerable share of action and power." When the especial disorder of the alimentary canal and liver appears great, depletion should be employed with more caution, and only in such a manner as "to keep the inflammatory actions within certain safe bounds, till the faulty state of the constitution can be corrected, or to conduct the disease through the stages which it naturally passes." To remove the disorder of the prima via, Dessault recommended the tartrite of antimony: Mr. James prefers the "common purging mixture, with such a proportion of tart. antimon. as may excite vomiting as well as purging; or, at all events, considerable nausea."

After recommending the removal of fecal accumulations, by calomel, &c. until the tongue begins to become clean; when the bowels may be moved by such "medicines as will give them some degree of tone, such as rhubarb and tinct. of senna; and at the same time saline draughts in effervescence, with antimonial wine, may be given." If these means do not stop the local mischief, nor remove the disorder of the prima via, and the disease increases, the author puts the question, "What measures are to be pursued? Is bark to be given?" His observations upon this very important point are worthy of notice: they meet our approbation.

"When there is much disorder of the prima via, bark is hardly a safe remedy; yet in many cases it is a very valuable one,—always, if it agrees. It is often worth while to try it; but the effect it produces must be carefully watched.

"We are directed to wait till the tongue cleans: this change often does not take place at all; and, when it does, it is, perhaps, immaterial what we do. I should rather judge by the state of the alvine discharges; for the presumption is, that, when the old fecal matters have been cleared away, and the secretions are copious and not very bad, bark may be borne with advantage; or when they are copious and cannot be improved by other means, that bark ought to be tried."

After recommending the bark to be given in the form of decoction with mineral acids, or with liq. ammon. acetat. and a warm tincture: he considers that benefit will be derived from a combination of the liq. ammon. acet. with confect. aromat., either with or without camphor, in such cases as may not be relieved by the exhibition of the bark. Soda-water, and wine in small quantity with arrow-root, "and negus, rather as a stimulus to the stomach than as a spur to the vascular system," are next spoken of, in terms of approbation. The advantage of cool and fresh air is then insisted on; after which the author passes to the consideration of the employment of sinapisms and fomentations to the feet, and blisters to the nape of the neck or between the shoulders. Mr. James introduces the subject of local applications with a few observations, tending to prove the service of such remedies to spontaneous erysipelas, even when occurring in the face, under certain states of the part affected and of the constitution. In the more acute local symptoms, he considers that leeches may be applied with advantage, with tepid evaporating lotions, or poultices. He recommends that an early exit may be given to matter, when it forms in the part. In this we are disposed to agree with Mr. James.

Some sensible remarks follow respecting this form of disease complicated with inflamed absorbents; in which he advises cold to be applied assiduously; blood-letting locally; the free use of incisions to the part, as proposed by Mr. Copland Hutchison; an elevated position of the limb; with evaporating poultices, and the free evacuation of matter or sloughs that may form in any situation. Respecting the manner of detecting these, and the mode in which they are to be evacuated, we can only say, that they deserve the attention of every surgeon. Some observations are also made upon the old practice of scarifications, as recommended in gangrenous inflammations by Quesnay and others; and upon the use of opium; and the means to be resorted to when the powers of the system begin to sink.

We cannot find space for the author's considerations on the treatment of the other species of erysipelatous inflammations; nor for any remarks on the remaining diseases of his classification. On expressing our opinion of the work generally, we should say, that the whole of the therapeutical part of it will fully reward the reader, though the language is frequently deficient in correctness, and the author's ideas are not always expressed with precision; whilst the pathological disquisitions (as might be understood from the early part of the review,) are deficient in some of the first requisites of sound reasoning. With those exceptions, it is creditable to the talents and the practical knowledge of the author.

